# Neuropsychological Findings in Gulf War Illness: A Review

**DOI:** 10.3389/fpsyg.2019.02088

**Published:** 2019-09-26

**Authors:** Mary G. Jeffrey, Maxine Krengel, Jeffrey L. Kibler, Clara Zundel, Nancy G. Klimas, Kimberly Sullivan, Travis J. A. Craddock

**Affiliations:** ^1^Institute for Neuro-Immune Medicine, Nova Southeastern University, Fort Lauderdale, FL, United States; ^2^VA Boston Healthcare System, Boston, MA, United States; ^3^Department of Clinical and School Psychology, Nova Southeastern University, Fort Lauderdale, FL, United States; ^4^Department of Clinical Immunology, Nova Southeastern University, Fort Lauderdale, FL, United States; ^5^Miami VA Medical Center, Miami, FL, United States; ^6^Department of Environmental Health, Boston University School of Public Health, Boston, MA, United States; ^7^Department of Psychology and Neuroscience, Nova Southeastern University, Fort Lauderdale, FL, United States; ^8^Department of Computer Science, Nova Southeastern University, Fort Lauderdale, FL, United States

**Keywords:** Gulf War illness, neurotoxicant, neuropsychology, posttraumatic stress disorder, veterans, review

## Abstract

This review paper summarizes the accumulation of research investigating neuropsychological outcomes in veterans with Gulf War illness (GWI). Earlier research focused on Gulf War veterans (GW) who were deployed versus non-deployed, as well as those who were symptomatic versus asymptomatic, or compared neuropsychological test results to published norms. Further research became more sophisticated, investigating specific GWI criteria, as well as the result of neurotoxicant exposure and the relationship to possible neurocognitive outcomes. As the early research supported both psychological and physiological effects on GWI; current research as summarized in this literature review supports the presence of neuropsychological deficits, particularly in the domains of attention, executive functioning, memory, and motor functioning related to chemical exposures that can be exacerbated by comorbid mood-related conditions. The same test battery has not been used consistently making it difficult to compare results among studies. Therefore, researchers created a resource to provide recommendations for the recently listed Neuropsychological Tests for Common Data Elements (CDEs) for use in all future GWI studies. Future research is necessary to further understand patterns of neuropsychological test data and how these decrements may relate to immunological or other biological markers, and the impact of trauma from physical and psychological stressors. In conclusion, there is consistent evidence that GWI is characterized by neuropsychological decrements – with future research these findings may aid in the diagnosis and assessment of treatment trial efficacy of GW veterans.

## Neuropsychological Findings in GWI: a Review

Gulf War illness (GWI), also known as chronic multi-symptom illness (CMI; Fukuda et al., 1998), has impacted approximately a third of the veterans deployed to the 1990–1991 Gulf War (Research Advisory Committee on Gulf War Veteran’s Illnesses [RAC-GWVI], 2008; White et al., 2016). By definition, GWI includes self-reported cognitive complaints indicative of neuropsychological impairment (Fukuda et al., 1998; Steele, 2000). Research examining the neuropsychological profile of GWI is necessary given that cognitive problems remain one of the most prevalent and distressing symptoms of GW veterans (Smith et al., 2012; Yee et al., 2016). Early on these cognitive symptoms resulted in veterans being referred for neuropsychological evaluations soon after their return from deployment and spurred research in this area. There have now been 25 papers specifically comparing objective neuropsychological performance in GW veterans with different comparison groups (deployed vs. non-deployed, symptomatic vs. non-symptomatic veterans, toxicant vs. non-toxicant). Here we present an overview of all 25 papers and describe the trajectory of research sophistication over time.

Neuropsychological findings in veterans with GWI have varied because of the use of different comparison populations (i.e., asymptomatic versus symptomatic GW veterans, different GWI cohorts) and different neuropsychological test batteries. A recent meta-analysis of the neuropsychological decrements associated with GWI provided some clarity to the question of which neuropsychological decrements were present in GW veterans and which tests were most sensitive to identifying these decrements. This was accomplished by combining multiple study results and using aggregate data when three or more studies used the same neuropsychological test. Studies were added into the meta-analysis when GW veterans served in the war from 1990 to 1991, had neuropsychological results reported in a manner conducive to meta-analysis, when comparison groups were deployed versus non-deployed or ill versus non-ill veterans and contained a unique sample (Janulewicz et al., 2017). The meta-analysis showed that the neuropsychological domains of visuospatial abilities, attention/executive functioning, and learning/memory were significantly different in GW veterans compared with two other comparison groups (Janulewicz et al., 2017). These findings remained significant when study results were adjusted for possible effects of publication bias (Janulewicz et al., 2017). In addition, analyses indicated that the following specific tests were most sensitive in discriminating between cohorts, including Block Design from the Wechsler Adult Intelligence Scale- Third Edition (WAIS-III; Wechsler, 1997), the Trail Making Test (Reitan, 1992), the Continuous Performance Test (CPT; Letz, 1991), and the California Verbal Learning Test (CVLT). The focus of the meta-analysis was not on the methodological strengths and weaknesses of the papers across the range of publications, but rather was to combine results to increase power and effect size across studies. These statistical findings were then used to determine which neuropsychological tests were most sensitive to GWI and were recommended in the recently listed neuropsychological component of the Common Data Elements (CDEs) for use in GWI studies^1^.

Recently, through the Congressionally Directed Medical Research Program (CDMRP) Gulf War Illness Research Program (GWIRP), a collaborative effort of GWI researchers was conducted to identify the CDEs or sensitive measures of cognitive functioning to guide future research and treatment trial efficacy (Gulf War Illness Research Program [GWIRP], 2019). Measures are presented in Table 1. These tests were chosen based on their sensitivity in distinguishing between groups in three or more prior studies with GW veterans. These tests were recommended so that future studies can compare biomarker and treatment trial outcomes between studies in a consistent manner and to use tests that are known to be sensitive to GWI.

**TABLE 1:** Gulf War illness common data elements module: neuropsychological test measures.

**Supplemental – Highly Recommended**
Word Reading Subtest of the Wide Range Achievement Test (WRAT-4) – (Wilkinson, 1993).
Continuous Performance Test-3 (CPT) – (Conners, 2014)
Wechsler Adult Intelligence Scale-IV (WAIS-IV) – (Weschler, 2008) Recommended tests: Digit Spans, Block Design
Profile of Mood States (POMS) – (McNair et al., 1971)
Davidson Trauma Scale (DTS) – PTSD - (Davidson et al., 1997).
Delis-Kaplan Executive Function System (D-KEFS) – (Delis et al., 2001) Recommended modules: Color-Word-Interference Test, Trail Making Test, Verbal Fluency
California Verbal Learning Test – Second Edition (CVLT-II) – (Delis et al., 2000)
Rey-Osterrieth Complex Figure Test (RCFT) – (Meyers and Meyers, 1995).
**Supplemental**
Finger Tap Test – (Reitan and Wolfson, 1993)
Grooved Pegboard Test – (Matthews and Klove, 1964)
Hopkins Verbal Learning Test (HVLT-R)^∗^ - (Brandt and Benedict, 2001)
Brief Visual Memory Test (BVMT)^∗^ - (Benedict, 1997)
PTSD Checklist for DSM-5 (PCL-5) – (Weathers et al., 1993)
Center for Epidemiological Studies Depression Scale (CES-D) – (Radloff, 1977)
Clinician Administered PTSD Scale (CAPS-5) – (Blake et al., 1990)
Structured Clinical Interview for DSM-5 (SCID-5) – (First, 2015)

*^∗^ denotes multiple test versions available for treatment trial use.*

In addition to facilitating neuropsychological outcomes research, researchers have also been learning more about the potential risk factors leading to objective neuropsychological decrements in GW veterans, including exposure to neurotoxicants (e.g., pesticides, nerve agents, and pyridostigmine bromide [PB] anti-nerve gas pills) as well as exposure to traumatic events during the war. These risk factors also include a history of mild traumatic brain injury and psychological trauma (Posttraumatic stress disorder (PTSD or mood disorder) (Sullivan et al., 2003, 2018; Yee et al., 2016, 2017; Janulewicz et al., 2017; Chao and Zhang, 2018).

More recent studies have also focused on biomarkers that are etiologically related to the neuropsychological deficits in GW veterans. These include toxicant induced neuroinflammation as well as war-time stressors (Brimacombe et al., 2002; Sullivan et al., 2003). Relevant to these factors are the rodent studies showing increased neuroinflammation when neurotoxicants were combined with simulated war-time stressors in the models (O’Callaghan et al., 2015; Ashbrook et al., 2018; Koo et al., 2018).

## Review of General Neuropsychological Findings

The neuropsychological literature has been reviewed multiple times by the RAC-GWVI (Research Advisory Committee on Gulf War Veteran’s Illnesses [RAC-GWVI], 2008, 2014; White et al., 2016). Additionally, review papers were published between 2000 and 2009, including Axelrod and Milner (2000), Vasterling and Bremner (2006), and White et al. (2016). More recently, a meta-analysis of the research data was published (Janulewicz et al., 2017). The current paper reviews the methodological strengths and limitations of the neuropsychological outcome studies to date. This includes 25 papers of which 14 were included in the meta-analysis. All 25 papers included assessments with validated neuropsychological instruments and were not case studies. Table 2 illustrates the increased sophistication in the field over time regarding case definitions and sensitive comparison groups and subsequent progress in understanding GW veterans’ neuropsychological profiles.

**TABLE 2:** Review papers on neuropsychological outcomes.

**References**	**Summary and recommendations**
Axelrod and Milner, 2000	• Concluded that methodological issues limited the ability to understand the data. • Recommended that future studies include more sophisticated cohort comparisons, including exposure data.
Vasterling and Bremner, 2006	• Concluded that there was no clear pattern in neuropsychological outcomes and insufficient neuroimaging evidence to draw conclusions at this point. • The impact of mood and the discrepancy between subjective reports and objective measurements made it more difficult to determine the etiology of any deficits observed. • Recommended that results need replication, objective measures of exposure should be used when applicable, baseline data should be used to investigate pre-existing vulnerabilities. • Future research should be built on more complex models that incorporate individual vulnerabilities, environmental factors and their physiological and emotional consequences and immunologic functioning.
Research Advisory Committee on Gulf War Veteran’s Illnesses [RAC-GWVI], 2008	• Concluded that symptomatic veterans have a subtle “sub-clinical” CNS damage. This included deficits in attention, executive function, memory, visuospatial skills, psychomotor functioning, and mood. • Recommended that analyses of veteran subgroups, i.e., those with more pronounced cognitive deficits and those with differing exposure histories, would be most informative.
White et al., 2016	• Concluded that GW exposures are associated with decrements in cognitive function. • Future research should investigate the mechanisms and etiology of GW health problems so that biomarkers of exposure and illness may be discovered.
Janulewicz et al., 2017	• Concluded with meta-analytic methods that GW deployment is associated with deficits in visuospatial, attention, executive function, and learning and memory but not simple motor function. • Future research developing treatments or investigating biomarkers of GWI should include neuropsychological outcomes in the domains of visuospatial, attention and executive function, and learning and memory. Particularly, Block Design, Trail Making Test, Digit Span, and CVLT, were sensitive measures to use with veterans with GWI.

Axelrod and Milner (2000) summarized the neuropsychological literature to date and reported that methodological problems limited the ability to understand the data. It was recommended that neuropsychological literature would be better served by including not only analyses based on normative data, non-deployed control comparisons, or self-reported medical concerns, but by also including relative risk for neurotoxicant exposures and hypothesis driven data collection.

Vasterling and Bremner (2006) found in their review of the literature, that there was no clear pattern in neuropsychological outcomes. Additionally, the impact of mood and the discrepancy between reported symptoms and objective performance made it more difficult to elucidate etiology of any deficits that were found.

In addition, the Research Advisory Committee on Gulf War Veteran’s Illnesses [RAC-GWVI] (2008) review concluded that symptomatic veterans had subtle “sub-clinical” CNS damage. This included deficits in attention, executive function, memory, visuospatial skills, psychomotor functioning, and mood. The RAC-GWVI Committee recommended that analyses of veteran subgroups, i.e., those with more pronounced cognitive deficits or those with differing exposure histories, would be most informative. When the general data regarding these studies were reviewed by the Institute of Medicine (Institute of Medicine [IOM], 2006), it was determined that overcorrecting for mood may have diminished the power to detect differences in neuropsychological variables in some prior studies.

White et al. (2016) concluded that exposures were associated with decrements in cognitive functioning in GW veterans and future research should investigate the mechanisms and etiology of GW health problems so that biomarkers of exposure and illness may be identified. In the recent GW meta-analysis by Janulewicz et al. (2017), it was reported that there were difficulties assessing domain specific findings given the sparse information reported in included studies, and the overlap between studies that prevented a more diverse sample. In addition, data were too limited to assess toxicant exposure in relation to neuropsychological deficits. Even with limitations across studies, it was found that deployed GW veterans and symptomatic GW veterans demonstrated levels of cognitive impairment, particularly in visuospatial abilities, attention/executive functioning, and learning/memory domains.

## Subjective Memory

Subjective memory has long been one of the most reported and debilitating symptom complaints of GW veterans. However, it has been unclear if this relates to objective memory deficits vs. attentional variability or the fatigue symptoms and sleep difficulties of those with GWI. It may also be that one-time objective neuropsychological testing in a quiet room does not fully capture functional memory concerns. The three studies that have addressed this topic to date include Binder et al. (1999), Lindem et al. (2003b), and Chao (2017).

Binder et al. (1999) incorporated measures of subjective cognitive complaints (e.g., Symptom Check List-90- Revised [SCL-90-R; Derogatis, 1992]) and affective distress (e.g., Beck Depression Inventory [BDI; Beck and Steer, 1993], Beck Anxiety Inventory [BAI; Beck et al., 1988]) in addition to a computerized test battery (Anger et al., 1996). With a sample of 100 symptomatic GW veterans, results showed higher correlations between subjective memory complaints and affective distress versus between subjective memory complaints and objective neuropsychological results. Therefore, Binder et al. (1999) concluded that affective distress was a necessary component of GW evaluations and provided additional explanation for worse cognitive outcomes in some GW veterans.

Lindem et al. (2003b) studied the relationship between neuropsychological symptom reporting and outcomes on objective tests in GW veterans. Symptom reporting was done with the Expanded Health Symptom Checklist (HSC, Proctor et al., 1998) which included five neuropsychological symptoms (e.g., difficulty concentrating, difficulty learning new material, forgetfulness, memory lapses, and confusion). Based on responses, participants were divided into groups of no complaints, a moderate level of complaints, and a high level of complaints. The researchers predicted that higher endorsement of neuropsychological symptoms would be associated with poorer performance on measures of attention and memory. Mood–related diagnosis was assigned using the following measures: Structural clinical interview for DSM (SCID; Spitzer et al., 1990), Clinical-Administered PTSD Scale (CAPS; Blake et al., 1990), the Mississippi Scale for Desert Storm, and Brief Symptom Inventory (BSI; Derogatis, 1993). Analyses were conducted to evaluate the ability of neuropsychological performance to categorize those with no, moderate, or high neuropsychological self-reported symptoms while controlling for covariates. Analyses indicated that subjective complaints did not show a pattern consistent with predicted performance on cognitive domains; however, they were more associated with mood complaints, which aligned with findings in Binder et al. (1999). Veterans with high levels of neuropsychological symptoms also reported tension, fatigue, confusion, and decreased vigor on the Profile of Mood States (POMS). Therefore, researchers concluded that these deficits are best measured by both objective neuropsychological testing and mood assessment to elucidate a clinical picture of GWI.

More recently, Chao (2017) conducted a study aimed at examining how subjective memory complaints (1 query of difficulty remembering) correspond with the likelihood of objective test results using the CVLT-II with a sample of 428 deployed GW veterans. Chao (2017) found significant impairment in verbal learning, retention, and recall in veterans with subjective complaints, even when accounting for age, sex, years of education, and mood-related diagnoses (e.g., major depressive disorder [MDD], PTSD, and anxiety). However, those with subjective memory complaints were more likely to have a PTSD diagnosis. Regression analyses also demonstrated poorer retention in association with subjective memory complaints. These results contrast with previous research (Binder et al., 2001; White et al., 2001) that did not find a connection between subjective complaints and objective impairment. Chao (2017) concluded that subjective memory complaints are sensitive to neuropsychological deficits and, as subjective memory complaints are linked to dementia risk, a necessary component of GW neuropsychological assessment.

These three studies did not show consistency in regard to objective tests (Table 3). Binder et al. (1999) and Lindem et al. (2003b) found results that linked subjective complaints to more mood-related factors, whereas Chao (2017) found evidence of objective memory impairment with subjective complaints. Given the discrepancy between subjective complaints and objective test performance, more validation research is needed with tests sensitive to memory impairment in GW veterans as delineated in the CDE protocol (Table 1). Also, none of these studies used the same subjective question of memory functioning making comparisons with objective measures difficult. Future studies should incorporate a validated subjective measure of cognitive functioning such as the Everyday Cognition Scale. In addition, careful use of statistical measures must be implemented to understand the unique contribution that mood and cognitive factors play in neuropsychological performance.

**TABLE 3:** Neuropsychological studies with Gulf War veterans.

**References**	**Group (N)**	**Neuropsychological Tests**	**Strengths**	**Limitations**	**Conclusions**
Goldstein et al., 1996	29 GWV, 39 non-veterans	WAIS-R Information WAIS-R Similarities WAIS-R Digit Span WAIS-R Arithmetic WAIS-R Digit Symbol WAIS-R Picture Completion WAIS-R Block Design COWAT Incidental Memory Verbal Associative Learning Short-Term Memory Symbol Digit Learning Trail Making Test CPT Grooved Pegboard	– GWVs compared to demographically matched controls on neuropsychological tests – Test battery included some measurements sensitive to GWI (i.e., Block Design)	– No case designation based on GWI – Limited measurement of mood	– Found that GWVs performed worse on impairment index in compared to controls – However, the level of impairments (1 SD) was not consistent with subjective cognitive complaints – Effect size Cohen’s d calculation^∗^ show small effect for Trails B (*d* = 0.25) and pegboard dominant (*d* = 0.18).
Axelrod and Milner, 1997	44 male GWV from Army Guard unit	Reitan-Indiana Aphasia Screening Test WAIS-R AVLT Stroop^∗^ Trail Making Test WMS-R Finger Tapping Grooved Pegboard^∗^ Grip Strength COWAT Category Fluency PIAT-R WCST	– Veterans compared via normative data and grouped by both objective (i.e., Grooved Pegboard, Stroop) and subjective (i.e., health complaint) measures	– Small sample size – Lack of correction for Type 1 error – Volunteer sample – No hypotheses	– No evidence of deficits – Differences in subjective complaints in psychological measures – Effect size Cohen’s d calculation^∗^ show large effect for Trails B (*d* = 1.28), and small/medium effect for motor tests (*d* = −0.63 to −0.48)
Hom et al., 1997	26 GWV with Haley Syndromes, 10 GWV and 10 non-deployed veteran controls	WAIS^∗^ Halstead Category Test^∗^ Tactual Performance Test Seashore Rhythm Test Speech-Sounds Perception Test Finger Oscillation Test Trail Making Test^∗^ Reitan-Indiana Aphasia Screening Examination, Reitan-Klove Sensory Perceptual Examination Reitean-Klove Lateral Dominance Examination Reitan Word Finding Test WMS-R^∗^ WRAT3^∗^	– Matched GWV group – GWI criteria used with a factor derived technique	– Small sample size – Limitations of those with fitting factor criteria – Multiple hypothesis testing Initial differences between control group and cases	– Differences between GWI and GWVs in global neurocognitive functioning – Cohen’s d calculation^∗^ showed a large effect size for Block Design (*d* = −1.57) and a medium effect size for Trail Making Test- Trail B (*d* = 0.69) – Psychological responding was consistent with other medical patients
Sillanpaa et al., 1997	49 GWV from a single Army reserve military police unit	NES-2 CPT^∗^ Grip Strength Grooved Pegboard^∗^ Neurological Screen Fingertip Number Writing perception^∗^ WCST^∗^ AVLT WAIS-R ^∗^	– Examiner blind to participant’s medical history – Exposure to toxins measured via self-report – Models tested to mimic GWI and psychological functioning	– Small sample size – Low variance and range in scores – Multicollinearity problems present	– Psychological model accounted for neuropsychological performance with a R^2^ of at least a 0.03 (at or above a small effect) for all domains – At least a small effect for exposure and symptoms seen in nearly all domains – Cohen’s d calculation^∗^ showed a medium effect for motor functioning (*d* = 0.76)
Vasterling et al., 1998	43 GWVs: 19 with PTSD and 24 without	Letter Cancelation Stroop CPT^∗^ WCST WAIS-R Digit Span WAIS-R Arithmetic^∗^ Rey-AVLT^∗^ CVMT^∗^	– Investigated GWVs with PTSD	– Small sample size – Lack of comparison sample of participants with differing mental disorders – Unable to manipulate trauma exposure	– Veterans with PTSD had deficiencies in sustained attention, mental manipulation, information acquisition, and retroactive interference
Anger et al., 1999	66 GWV with unexplained symptoms from WA and OR, 35 GWV controls	Behavioral Assessment and Research System (BARS): Simple Reaction Time^∗^ Selective Attention Test Digit Span^∗^ Symbol Digit^∗^ Serial Digit Learning ODTP^∗^	– Compared those with GWI with controls – Physician blind to participant status	– Volunteer sample Self-selection bias	– Specific problems demonstrated in processing speed – Individuals compared based on processing speed differences also found that those with slower processing speed had deficits in memory and attention
Binder et al., 1999	100 GWV with unexplained symptoms	CPT ODTP^∗^	– Investigated self-report of cognitive ability and affective distress in conjunction with objective cognitive performance	– Cognitive measures may lack sensitivity	– Subjective complaints associated with psychological distress over objective cognitive performance
Bunegin et al., 2001	8 symptomatic GWV, 8 GWV controls	NES-2: Hand-Eye Coordination Simple Reaction Time Visual Digit Span Forward and Backward^∗^ Horizontal Addition Pattern Memory^∗^ Switching Attention^∗^	– Compared symptomatic GWVs with non-symptomatic GWVs – Investigated blood flow	– Small sample size – Less sensitive measures used	– Symptomatic GWVs had worse performance in memory and executive function tasks – Exposure to acetone also impacted cognitive performance in GWVs – Cohen’s d calculation^∗^ showed a small effect size for CPT, reaction time (*d* = −0.14)
Lange et al., 2001	48 symptomatic GWV, 39 GWV controls	NES^∗^ PASAT^∗^ WAIS-R Digit Span^∗^ CVLT RCFT Trails Making Test Category Test^∗^ Judgment of Line Orientation Test WAIS-R Block Design Grooved Pegboard	– Compared those with GWI with matched controls	– Volunteer sample of health-care seeking veterans – Small sample size CFS sample – Unequal cells comparisons	– Impairment found in attention (*R*^2^ = 0.12– 0.19) and executive functioning tasks (R^2^ = 0.07) even after controlling for mood – Cohen’s d calculation^∗^ showed a large effect size for CPT reaction time (*d* = 0.85).
White et al., 2001	193 GWV, 47 Germany deployed veterans	WAIS-R CPT Trail Making Test PASAT WCST Digit Span CVLT^∗^ WMS-R^∗^ Finger Tapping Purdue Pegboard POMS^∗^ TOMM	– Compared deployed and non-deployed veterans – Detailed account of toxin exposure – Stratified Random sample	– TOMM scores evidenced possible poor effort in some participants – Multiple comparisons	– Initially, mood was only significant with adjustment for multiple comparisons – Comparing those with and without exposure, had worse performance in short term memory (R^2^ = 0.315– 0.399), attention (R^2^ = 0.381), and mood ((R^2^ = 0.202– 0.315) – Cohen’s d calculation^∗^ showed small effect sizes for all neuropsych tests (*d* = −0.47 to 0.22
David et al., 2002	209 British GWV, 132 non-deployed era veterans	WAIS-R NART WAIS-III Letter Sequencing PASAT SART Stroop Trail Making Test WMS-R Purdue Pegboard	– Compared Gulf War deployment and medical status against other deployments (Bosnia) and controls – Stratified Random sample Blind raters Statistical analyses	– Cross-over effects Self-report symptoms	– Significance only found in PTSD measure – Cohen’s d calculation^∗^ showed a large effect size for Block Design (*d* = −2.53)
Lindem et al., 2003a	193 GWV, 47 Germany deployed veterans	WAIS-R Information subscale^∗^ WAIS-R Digit Span^∗^ WMS-R Digit span CPT^∗^ Trail Making Test WCST PASAT^∗^ Finger Tapping^∗^ Purdue Pegboard^∗^ WAIS-R Block Design WMS-R Verbal Paired Associate Learning CVLT^∗^ Visual Reproduction^∗^ POMS^∗^	– Investigated PTSD in relation to exposure to chemical agents – Investigated symptom severity – Better generalization with use of overall cohorts in Gulf War – Large sample size	– Correlational analyses – Lack of baseline performance or known preexisting conditions	– PTSD symptoms severity correlated with greater deficits in a wide array of neuropsychological measures in GW deployed veterans (Partial *R*^2^ = 0.02– 0.10) – CBW exposure and PTSD severity in GWVs associated with deficits in sustained attention (Partial *R*^2^ = 0.0004– 0.0015), motor speed/motor coordination (0.0000–0.0007)
Lindem et al., 2003b	193 GWV, 47 Germany deployed veterans	WAIS-R Information subscale WAIS-R Digit Span WMS-R Digit span CPT Trail Making Test A Trail Making Test B WCST PASAT Finger Tapping Purdue Pegboard WAIS-R Block Design WMS-R Verbal Paired Associate Learning CVLT Visual Reproduction POMS^∗^	– Investigated GWVs discrepancy between subject complaints and objective performance	– Multiple comparisons	– Subjective complaints more associated with mood symptoms
Lindem et al., 2003c	58 GWV and 19 Germany-deployed veterans	WAIS-R Information subscale WAIS-R Digit Span WMS-R Digit span CPT Trail Making Test^∗^ WCST^∗^ PASAT Finger Tapping Purdue Pegboard WAIS-R Block Design WMS-R Verbal Paired Associate Learning^∗^ CVLT^∗^ Visual Reproduction^∗^ TOMM^∗^ POMS	– Investigated motivation in GWVs	– Small sample size – Difficult to ascertain the reason behind lower TOMM scores – Low amount of those with low TOMM scores	– Variability was seen in those with lower TOMM scores particularly in attention, executive functioning, and memory
Proctor et al., 2003	Danish GWVs (215), comparing deployed (143) and non-deployed veterans (72)	WAIS-R Information CPT Trail-making Test Wisconsin Card Sorting Test Purdue Pegboard WAIS-R Block Designs California Verbal Learning Test WMS Visual Reproductions POMS^∗^ TOMM	– Blind to categorization of “higher or lower” symptom status during all phases of recruitment, testing, and interviewing – Differences in deployment missions between Danish and American groups	– Significant mean age difference between deployed (38.8 years) and non-deployed (34.8 years) – Self-report of exposure	– Evidence of increased mood complaints related to GW service – no significant domain-specific evidence of CNS dysfunction was found – No associations between reported GW Environmental exposures related to the Danish GW deployment mission and objective measures of cognitive functioning were observed
Sullivan et al., 2003	207 treatment seeking GWV (120 referred for neuropsych evaluation), 53 treatment seeking non-deployed veterans	WAIS-R Information WAIS-R Digit Span^∗^ Trail Making Test NES CPT Stroop Test Paced Auditory Serial Addition Test Wisconsin Card Sort Test^∗^ CVLT WMS-R Paired Associate Learning WMS-R Visual Reproductions^∗^ Hooper Visual Organization Test WAIS-R Block Design^∗^ Finger tapping Purdue Pegboard RCFT^∗^ POMS^∗^ TOMM	– Investigated deployment, treatment seeking, use of pyridostigmine bromide (PB) and PTSD on cognitive functioning – Matched by control group that was also treatment seeking	– Self-report of exposure – Sample size small for comparisons	– GW deployed worse than controls on attention, visuospatial skills, visual memory, and mood – PB use in GWVs worse in executive system tasks – GWVs with PTSD versus those without PTSD showed no differences – Cohen’s d calculation^∗^ showed a large effect sizes for block design and digit span forward (*d* = −2.43 to −1.00), small effect sizes for Trails A and B, digit span backward, CVLT, WMS, immediate recall, and finger tapping (*d* = −0.090 to 0.43), and a medium effect size for WMS, delay recall (*d* = −0.55).
Vasterling et al., 2003	72 GWVs deployed and 33 non-deployed GWVs	WAIS-R Digit Span WCST AVLT CVMT Purdue Pegboard WAIS-R Information	– Selection of a non-treatment seeking group of GWVs – Comparison of deployed GWVs to a group of GWVs mobilized but no deployed – Use of olfactory and neurocognitive measures with demonstrated sensitivity to neurotoxic exposures	– Sample was regionally recruited	– No evidence that performance on olfactory or neurocognitive measures were related to war-zone duty or to self-reported exposure to GW toxicants – Symptoms of emotional distress were positively correlated with self-report of health and cognitive complaints
Proctor et al., 2006	140 Army GWV with modeled estimates of nerve agent exposure	CPT Trail Making Test WAIS-R Digit Span WCST Finger Tapping Purdue Pegboard^∗^ WAIS-R Block Design^∗^ CVLT WMS-R verbal paired associate learning WMS visual reproduction	– Stratified random sampling – Examined performance by exposure to sarin and cyclosarin – Sample was unaware of sarin and cyclosarin components, analyses were conducted a prior to exposure knowledge	– Etiology undetermined given the risk of another illness between exposure and measurement (i.e., no baseline health information) – Limited objective information about exposures	– Exposure associated with poor fine psychomotor dexterity (*d* = 0.44) and visuospatial abilities (*d* = 0.43)
Barrash, 2007	301 GWV, 99 era veterans deployed elsewhere	WAIS-III Similarities^∗^ Block Design^∗^ Digit Symbol Digit Span North American Reading Test – Revised Starry Night Test COWAT AVLT^∗^ Benton Visual Retention Test^∗^ RMT-Words and Faces^∗^ Stroop Grooved Pegboard^∗^	– Study of effort and neurocognitive performance in GWVs –Grouped by credible or non-credible impairment	– Small sample of non-credible group –Decreased statistical power – Lack of measures investigating reason behind low effort	– Non-credible impairment associated with more variability in tests and worse emotional/cognitive functioning
Wallin et al., 2009	41 GWVs: 25 with GWI and 16 controls	WRAT reading Block Design Trail Making Test CVLT Pegboard	– Stratified random sampling – Used GWI criteria to divide groups	– Small sample size – Gap between deployment and time of study – Multiple analyses	– Differences only seen in mood and health measures – Cohen’s d calculation^∗^ showed a medium effect size for block design, Trails B, and CVLT long delay, (*d* = −0.73 to 0.51), and a small effect size for WRAT reading, Trails A, and Pegboard (*d* = −0.13 to 0.39).
Toomey et al., 2009	1061 deployed GWV and 1128 non-deployed GWV	WAIS-III Digit Span Trail Making Test^∗^ PASAT CPT^∗^ WCST CVLT^∗^ RCFT^∗^ Finger Tapping^∗^ Purdue Pegboard^∗^ TOMM WRAT-III	– Investigated differences in deployment, toxin exposure, and GWI status – Large sample size, stratified random sampling method – Use of factor analysis – Use of Khamisiyah exposure data	– Low study participation rates – Cross-sectional design – Neuropsychology raters were not blind to condition	– Deployed veterans had worse performance on motor speed (OR = 2.35) and sustained attention (OR = 2.64) – Those with Khamisiyah exposure showed poor motor speed after controlling for mood – Cohen’s d calculation^∗^ showed small effect sizes for all neuropsych tests (*d* = −0.09 to 0.06).
Chao et al., 2010	40 GWV with a history of DOD notified sarin cyclosarin exposure risk and 40 non-exposed matched GW veteran controls	CPT Trail Making Test WAIS-III Digit Span Short Category Test COWAT Grooved Pegboard WAIS-III Digit Symbol, matching WAIS-III Block Design WAIS-III Verbal Comprehension Index CVLT-II WMS-III Logical Memory BVMT-R TOMM^∗^	– Used matched cohort sample – Use of Khamisiyah exposure data	– Lack of information regarding the unit and rank of veterans – Lack of information regarding symptom severity (i.e., CMI, smoking status, head injuries) – Lack of cumulative exposure for all GW veterans – Plume estimates only by unit	– No differences in cognitive measures after controlling for poor effort (i.e., failure of TOMM). – Cohen’s d calculation^∗^ showed a small effect sizes for all neuropsych tests (*d* = 0.22 to 0.26).
Chao et al., 2011	64 sarin and cyclosarin exposed GWVs and 64 “matched” unexposed GWVs	CPT^∗^ WAIS-III Digit Span^∗^ Trail Making Test Short Category Test CVLT-II^∗^ Grooved Pegboard TOMM	– Used matched controls to compare structural and functional differences in veterans with suspected neurotoxicant exposure – Use of more sensitive MRI (4T) – Use of some sensitive tests for neuropsychological and mood outcomes	– Lack of information regarding veteran’s unit, severity of GWI symptoms, smoking status, or history of head injury – Neurotoxicant exposure measured at unit over individual level	– Reduced gray matter and white matter in exposed veterans which was linked to neurotoxicant exposure – Exposed veterans made more omission errors and had slower responses times; omission errors was also linked to neurotoxicant exposure – Cohen’s d calculations^∗^ showed a medium effect size for Trails A (*d* = −0.64), and small effect sizes for CPT, Trails B, CVLT, and pegboard (*d* = −0.36 to 0.38).
Chao et al., 2016	136 GWVs: 106 who reported hearing chemical alarms sound	WAIS III Block Design^∗^ Digit Span^∗^ CVLT		– Had to rely on self-reports of deployment-related exposures – Lack of pre-GW measurements of brain structure and function – Small sample size – Lack of a non-deployed GW-era veteran control group	– Self-reported frequency of hearing chemical alarms was inversely associated with and significantly predicted performance on the Block Design visuospatial task. – This effect was partially mediated by the relationship between hearing chemical alarms and lateral occipital cortex volume.
Chao, 2017	428 deployed GWVs: 272 which met CDC criteria for CMI	CVLT-II^∗^	– Tested verbal memory with GWVs presenting with subjective memory complaints – Large sample size	– Only measured one domain to control for Type 1 error	– Worse performance on verbal memory associated subjective complaints over and above mood, however, there was higher endorsement of PTSD symptoms
Sullivan et al., 2018	159 GW-deployed preventative medicine personnel who had varying levels of pesticide exposure	WAIS-III information subtest Boston Naming Test Trail Making Test^∗^ CPT^∗^ WCST Finger Tapping Grooved Pegboard HVOT RCFT^∗^ Stanford-Binet Copying Test CVLT II POMS^∗^ TOMM	– Grouped veterans by exposure (low/high) to PB and pesticides – Sample had sophisticated knowledge of exposure as they were part of the medical team	– Multiple analyses – Exposures of PB and pesticide may be correlated – Classifications of groups based on self-report	– High pesticide/high PB had worse information processing speed, attention (i.e., errors), visual memory, and increased mood complaints

*See original journal articles in first column for test references. ^∗^Denotes significance of *p* < 0.05.*

## Neuropsychological Performance as Compared by Normative Data

The following two early studies (Axelrod and Milner, 1997; Sillanpaa et al., 1997) examined those deployed in the GW in comparison to normative data. Axelrod and Milner (1997), tested 44 male GW veterans on a comprehensive neuropsychological exam (Table 3). Compared to normative data, deficits were found on only a motor test; Grooved Pegboard and a test of executive function; Stroop Color and Word Test (Matthews and Klove, 1964; Heaton et al., 1992). The researchers attributed the neuropsychological issues to elevations on selected subtests of a personality measure the Minnesota Multiphasic Personality Inventory Second Edition (MMPI-2; Graham, 1990). However, Janulewicz et al. (2017) found through examination of effect sizes, that cognitive flexibility as measured by the Trail Making Test- Trail B had a large effect size, while a small to medium effect was seen in motor tests, which may show some deficits that were masked by a small sample size. Other limitations were the lack of a control group (i.e., comparison to normative data collected from a non-military population), and lack of control regarding covariates of cognitive performance (i.e., age, gender, developmental history), and psychopathology (i.e., PTSD).

Sillanpaa et al. (1997) investigated neuropsychological and neurological functioning in 49 GW veterans from an Army Reserve Military Police unit. Each veteran completed personality and neuropsychological testing (Table 3). Neuropsychological performance was evaluated in comparison to normative data and models were created to test variables associated with a syndrome and to test variables associated with mood. The syndrome model included demographic factors, self-reported exposure to toxicants and a composite score of subjective complaint (i.e., composed of scores from the SCL-90-R and MMPI-2), and a clinical signs index (i.e., composite score of laboratory tests for liver and immune functioning or infection presence). The model of mood-related issues included indices of trait anxiety, subjective complaints, depression, and state anxiety. Results indicated that mood-related factors (i.e., anxiety, depression) accounted for more variance in neuropsychological performance measuring attention, motor coordination, and executive functioning in comparison to the syndrome model. However, limitations of the study included a small sample size and the use of a syndrome model that does not represent the current case criteria for GWI (i.e., CMI or Kansas GWI criteria).

These two studies (Axelrod and Milner, 1997; Sillanpaa et al., 1997) were similar in that they attributed more mood-altering factors to neuropsychological functioning as measured by the MMPI-2. However, comparison of effect sizes may point toward a trend in relatively impaired motor coordination and executive functioning.

## Deployment Status and Neuropsychological Findings

Goldstein et al. (1996) tested 21 GW deployed veterans with a battery of neuropsychological tests and compared their performance to results from 38 demographically matched non-military controls. Cognition was measured via an extended version of the Pittsburgh Occupational Exposure Test battery (Table 3). Psychological distress was measured via the SCL-90-R. An impairment index was composed of 14 total neuropsychological tests (Table 3). Differences were found in the overall impairment index with significantly poorer performance in deployed compared to the control group. When controlling for mood, the impairment index difference was no longer significant. Specific impairments were found on the Controlled Oral Word Association Test (COWAT) and the Continuous Performance Test (CPT) reaction time. Notable limitations of this study are the small sample size utilized as well as the use of matched controls from a non-military population. Effect sizes noted in the recent meta-analysis by Janulewicz et al. (2017) reported a small effect (0.25) in the Trail Making Test -Part B between the groups, while the Grooved Pegboard (dominant) score approached a small effect size (0.18).

White et al. (2001) performed neuropsychological testing and compared the outcomes in those with specific self-reported neurotoxicant exposures. Veterans (*n* = 240) were recruited from 2 deployed and one non-deployed cohorts (Proctor et al., 1998). Veterans underwent an environmental interview, mood surveys, a full neuropsychological test battery (Table 3) and a psychological diagnostic interview. Neuropsychological outcomes showed differences in CPT when mood covariates were not controlled for; however, no individual measure achieved statistical significance when controlling for mood. Of note, additional tests showed moderate effect sizes in measures of attention, executive, and motor function (Paced Auditory Serial Addition Test (PASAT), Wisconsin Card Sort Test (WCST), Trail Making Test- Part A, Purdue Pegboard) which suggest that those deployed in the GW had poorer cognitive performance.

David et al. (2002) investigated neuropsychological patterns among 341 veterans who served in the United Kingdom military forces. Out of 341 participants, 98 were designated “Gulf well,” 111 were designated “Gulf ill,” 78 were designated “Era ill” and 54 were designated “Bosnia ill.” David et al. (2002) assessed general functioning through a complete neuropsychological battery (Table 3). In regard to neuropsychological test performance, the GW ill group had poorer performance on WAIS-R Performance IQ, the digit symbol test, the Trail Making Test, and Sustained Attention to Response Task (SART) accuracy. After adjusting for the BDI score and multiple comparisons, no significant differences were found between healthy and GW ill on cognitive performance measures. David et al. (2002) found that the ill group had higher scores on the Mississippi Combat Related PTSD Scale. When testing the main effect of deployment, it was found that participants in the Gulf group had significantly lower Verbal IQ and Performance IQ scores (i.e., most notably, in Block Design) compared to the Era ill Group. Additionally, the Gulf ill group had the lowest pegboard performance compared to the other groups. After controlling for BDI scores, there were still significant differences in Verbal IQ and the Purdue Pegboard when comparing the Gulf ill group to other groups. However, these contrasts were not significant after adjusting for multiple comparisons. Therefore, David et al. (2002) concluded that there was no major neuropsychological impairment, but rather, more associations with mood related impairment in deployed veterans which may better account for poor performance on neuropsychological measures. However, by controlling these factors, they may have discounted the mood symptoms may have resulted from neurological impairment and/or neurotoxicant exposures. Additionally, before correction, there was indication that individuals who were GW ill may have difficulties associated with performance in Performance IQ, Digit Symbol, Trail Making Test- Part A and B, and SART errors. Additionally, GW ill was also associated with poorer performance in Verbal IQ, Performance IQ, Block Design, and the Purdue Pegboard. Furthermore, these studies highlight the importance of overcorrection for emotional symptoms that may lead to underestimating true neuropsychological deficit that can also lead to mood symptoms as stated by Institute of Medicine [IOM] (2006).

Lindem et al. (2003a) investigated neuropsychological performance in conjunction with chemical exposure and severity of trauma symptoms with a sample of 225 deployed and non-deployed participants. Participants were administered the CAPS to determine the level of trauma symptoms. In addition, the veterans underwent a full neuropsychological test battery White et al. (2001). Chemical exposure was assessed through self-report measures and a clinical interview. Results indicated that the severity of PTSD symptoms in the full sample after controlling for covariates was directly correlated with poorer performance in general intellectual ability, attention, motor, memory, and mood measures. In GW deployed veterans, partial correlations were significant for those with PTSD and worse performance on general intellectual ability, sustained attention, motor functioning, verbal learning, and all mood scales.

Proctor et al. (2003) studied neuropsychological measures in deployed (*n* = 143) and non-deployed veterans (*n* = 72) Danish GW veterans. Researchers compared groups across neuropsychological measures (White et al., 2001), controlling for age. It was found that there were significant differences for neuropsychological domains; such that individual tests of executive functioning and verbal memory showed poorer performance in the deployed veterans. There was significant difference on the POMS Fatigue and Confusion scales, with deployed groups reporting a moderate to high number of symptoms. Therefore, the researchers concluded that, as there was no connection between deployed and non-deployed groups on neuropsychological measures, there was no evidence in this study of toxicant exposure leading to neurocognitive deficits. Rather, mood related symptoms were more likely to be reported. However, this study was composed of Danish soldiers who were not exposed to combat and were not in chemical warfare areas indicating that they likely differed from other cohorts (e.g., British, American) given differential exposure to GW neurotoxicants (less endorsement of exposure to chemical warfare agents and no use of anti-nerve gas pills) and less trauma. However, further investigation of the effect sizes via Janulewicz et al. (2017) found small effects in the Trail Making Test (*d* = 0.22 to 0.31) and in a memory measure (CVLT; *d* = −0.32 to −0.20). Block Design approached a small effect as well (*d* = −0.18).

Sullivan et al. (2003) evaluated a sample of 260 veterans including GW deployed and seeking treatment (i.e., for cognitive or health symptoms) and a control group of GW non-deployed veterans seeking neuropsychological evaluations. All veterans underwent a neuropsychological battery (Table 3) in addition to a structured clinical interview. In comparison to non-deployed veterans, deployed veterans had worse performance in measures of attention, visuospatial skills, and visual memory. In addition, deployed veterans endorsed worse mood symptoms. Therefore, the researchers concluded that GW deployment led to the significant neuropsychological decrements. Effect size analysis performed by Janulewicz et al. (2017) found a small effect in Trail Making Test- Part A (*d* = 0.43), Trail Making Test- Part B (*d* = 0.36), CVLT Trials 1–5 (*d* = −0.26), CVLT short delay (*d* = −0.47), CVLT long delay (*d* = −0.42), CVLT recognition (*d* = −0.33), and WMS immediate recall (*d* = −0.55). A medium effect was seen in WMS delayed recall (*d* = −0.55). A large effect or higher was seen in Digit Span backward (*d* = −1.00), and Block Design (*d* = −2.43).

Toomey et al. (2009) conducted a study examining GW veterans (deployed *n* = 1061, and non-deployed 1,128) on several measures of neuropsychological performance. Veterans underwent a neuropsychological battery (White et al., 2001). Results indicated that deployed veterans performed significantly worse on a measure of attention flexibility (i.e., Trails A-B) in comparison to non-deployed veterans. The meta-analysis completed by Janulewicz et al. (2017) did not return any notable effect sizes.

Overall, these studies comparing deployed GW veterans to non-deployed veterans showed some consistency in relative impairment within major cognitive domains, including simple and sustained attention, complex tracking, working memory, acquisition and retention of information when simply comparing deployment status rather than symptomatic vs. non-symptomatic groupings.

## Symptomatic Vs. Non-Symptomatic Deployed GW Veterans and Neuropsychological Performance

Hom et al. (1997) first investigated symptomatic GW veterans (*n* = 26) in comparison to healthy GW veteran controls (*n* = 20) on neuropsychological and psychological measures (Table 3). Psychological functioning was measured using validated surveys and a clinical interview. Symptomatic veterans showed significantly worse performance on measures of overall brain function or derived composite scores from neuropsychological measures (Halstead Retain Impairment Index). In addition, symptomatic veterans showed greater impairment than controls on the Halsted Category Test and Trails Making Test- Part B (*d* = 0.69 per Janulewicz et al., 2017), indicating poor abstract reasoning and problem solving/flexibility; measures of executive functioning. Of note, Janulewicz et al. (2017) also found a large effect size for Block Design (*d* = −1.57) for this study. The researchers concluded that these results supported the presence of worse neuropsychological and mood functioning in veterans with GWI as classified by Haley syndromes (Haley et al., 1997). However, these researchers hypothesized that mood complaints were secondary to the physical dysfunction consistent with GWI symptoms and did not solely account for GWI presentation. This study exhibited several limitations including a small sample size.

Anger et al. (1999) investigated mood and neuropsychological differences in GW veterans with unexplained medical symptoms. Veterans underwent a medical examination conducted by a physician blind to case/control designation; controls were determined as those not endorsing any GW related symptoms. Symptomatic and non-symptomatic veterans (*N* = 101) completed a series of tests assessing psychological and neuropsychological functioning (Table 3). Anger et al. (1999) found statistically significant differences on neuropsychological testing only for the Oregon Dual Task Procedure (ODTP) computerized test measure after controlling for multiple comparisons. Using these results, researchers divided groups based on speed as “slow cases” and “other cases.” Consistent with performance on the ODTP, veterans in the “slow case” group showed slower responses than controls on Symbol Digit, Simple Reaction Time, Digit Span Forward, and Digit Span Backward. Therefore, Anger et al. (1999) reported slower neurobehavioral performance on digit recall tasks and increased psychological distress in those with GWI symptoms. However, slow performance was exhibited in a sub group of GW cases (“slow cases”). These “slow cases” also showed deficits in working memory, attention and response speed indicating a more severe subgroup. These results were also not otherwise explained by mood or PTSD and were consistent with the literature investigating deficits in those with organophosphate poisoning.

Storzbach et al. (2000) conducted a study investigating the performance of GW veterans with unexplained symptoms (*n* = 241) on psychosocial and neurobehavioral measures in comparison to a veteran control group (*n* = 113). In regard to the mood measures, there was a significant difference between groups in that symptomatic veterans were higher on nearly all mood measures with nearly all measures demonstrating a large effect size. Additionally, the case group endorsed worse physical, mental, and health-related functioning (SF-36), greater combat exposure scale measures, and PTSD symptoms. In regard to neuropsychological testing, the case group had worse performance on Symbol Digit and ODTP forced choice and forced latency scores with a small effect size (Smith, 1968; Binder, 1993). The researchers concluded that, as they found differences in psychosocial and cognitive tests, stress has a major role in GW symptoms as either a precursor or a result of the experienced symptoms. However, these conclusions are limited in that the researchers did not control for mood when investigating neuropsychological performance.

Storzbach et al. (2001) expanded upon these findings using the same measures to assess psychosocial and cognitive functioning in 239 symptomatic GW veterans and 112 control veterans. However, they identified a “slow group” using a modified cutoff as established by Anger et al. (1999). The slow group had worse performance in comparison to controls in all measures, except the Serial Digit Learning Test again indicating a more impaired subgroup.

Binder et al. (2001) investigated cognitive performance in symptomatic GW veterans (*n* = 94) as defined by chronic fatigue syndrome (CFS). Groups were divided based on CFS criteria (Fukuda et al., 1994) with 32 participants comprising the case group and 62 participants comprising the control group. Neuropsychological testing was conducted using the same battery described in Anger et al. (1999). Results indicated that those in the case group performed worse on reaction time ODTP latency, and ODTP number correct. Limitations of this study include the use of a computerized measure that may have been less sensitive then measures with an examiner and a shorter battery with less global neurocognitive implications and the classification of GWI as CFS.

Bunegin et al. (2001) built their hypothesis on the premise that GW symptoms are linked to CNS dysfunction. Previous research has shown that GW veterans experience cognitive issues and headaches from chemical odors (Bell et al., 1990; Miller and Prihoda, 1999) which is similar to transient ischemia. Therefore, researchers investigated cognitive performance and middle cerebral artery blood flow velocity (MCABFV) in both symptomatic (*n* = 8) and asymptomatic GW veterans (*n* = 8) when exposed to different air conditions (i.e., clean air, placebo acetone condition, and low levels of acetone). All participants were tested using NES-2 computerized assessment (Letz, 1991). The results of the study suggested that both symptomatic and asymptomatic GW veterans performed similarly in cognitive tests when comparing the performance across different air exposures. However, pooled data across conditions revealed significantly lower performances in measures of memory and executive functioning in symptomatic GW veterans. Additionally, there were statistically significant differences between asymptomatic and symptomatic GW veterans in MCABFV as symptomatic GW veterans demonstrated a depressed response across all conditions.

Lange et al. (2001) conducted a study examining symptomatic and healthy GW veterans on cognitive functioning; however, symptomatic was defined using established criteria for CFS and Multiple Chemical Sensitivity (Cullen, 1987; Fukuda et al., 1998). Additionally, Lange et al. (2001) identified and accounted for presence of PTSD and major depression in a group of 87 GW veterans (healthy controls = 39; GWI = 48). Both healthy and symptomatic GW veteran groups were administered tests sensitive to attention, concentration or information processing, verbal and visual memory, abstraction and conceptualization, visuo-perceptual and perceptual-motor functions, and fine motor functioning. Analyses found significant results in attention, concentration, and information processing, as well as abstraction and conceptualization. Tests reflecting attention and information processing as well as tests of abstraction and concentration were significantly different with symptomatic veterans showing worse performance than non-symptomatic controls. In addition, regression analyses were conducted controlling for mood outcomes; results indicated the symptomatic group remained significant on some tests (NES simple reaction time) but were no longer significant for other tests. Mood-related diagnoses were not correlated with performance on the CPT; therefore, case status was the only predictor and remained significant in symptomatic GW veterans. Lange et al. (2001) concluded that symptomatic veterans exhibited deficits on attention, concentration, and information processing over and above the impact of mood related disorders. Limitations of the study include using GWI terminology inconsistent with the field where current case criteria is determined using Kansas or CDC criteria rather than CFS and MCS.

Wallin et al. (2009) investigated neuropsychological performance in a small sample derived from the National Health Survey of GW veterans (Case group with CDC criteria = 25, Control = 16). Veterans underwent neuropsychological testing (Table 3) in addition to psychological testing. Wallin et al. (2009) found no significant differences between groups on neuropsychological testing. However, there were differences in GWI cases on measures of depression, somatic complaints, and anxiety. Wallin et al. (2009) concluded a stronger influence of psychological factors over neurological factors. Several limitations were present in this study including a small sample size. Correspondingly, an effect size analysis conducted by Janulewicz et al. (2017) found meaningful effects (>0.20) in Block Design (*d* = −0.73), Trail Making Test, Part-A (*d* = 0.39), Trail Making Test, Part-B (*d* = 0.51), CVLT, long delay (*d* = −0.66), Pegboard, dominant (*d* = 0.37) and Pegboard, non-dominant (*d* = 0.31) that would have shown significant differences in a larger study sample.

These eight studies had similar findings regarding cognitive domains when investigating symptomatic vs. non-symptomatic veterans indicating the more refined criteria than deployed vs. non-deployed. In the symptomatic groups there was consistency in attention deficits as measured Digit Span Forward in several studies (Table 3). Additionally, psychomotor speed as measured by Symbol Digit and Simple Reaction Time was sensitive to symptomatic veterans (Table 3). Finally, the most consistent finding regarding attention was in a measure of sustained attention – CPT, which was observed in several studies. Executive functioning was more variable with less consistency in test measures used. Therefore, there was few similarities between studies (i.e., no more than two studies had similar findings in Category Test and Trail Making Test- Part B). In regard to memory, several studies found memory impairment as measured by the CVLT-II (Table 1). These findings are supported by both imaging and animal models of memory. Regarding visuospatial functioning, there was consistency of results in that the symptomatic group showed worse performance on Block Design in multiple studies (Hom et al., 1997; Proctor et al., 2006; Chao et al., 2010). Finally, there was some consistency in motor coordination as measured by a Pegboard test in several studies (Proctor et al., 2006; Chao et al., 2010, 2011). In terms of mood measures, these studies did not have more than two studies that were consistent in mood results. However, health outcomes as measured by the SF-36 were different in the case symptomatic groups (Anger et al., 1999; Storzbach et al., 2000; Wallin et al., 2009).

These studies were able to demonstrate a stronger argument for neurological dysfunction given the clear operational definitions of symptomatic vs. non-symptomatic groups. However, these studies were still very diverse in regard to the measurement style (i.e., computer versus paper and pencil) and test battery and what determined ‘caseness.’ These studies also highlight the importance of using more objective measures of neurological biomarkers to make a stronger argument for behavioral and neurological connections. As noted before, future research using CDEs for case criteria and neuropsychological batteries would be highly beneficial given their sensitivity to changes in veterans with GWI as well as creating more consistency across studies.

## Neurotoxicant Exposure and Neuropsychological Performance

Several studies have assessed neuropsychological functioning in relation to neurotoxicant exposures during the war including sarin, pesticides and pyridostigmine bromide (PB) anti-nerve gas pills. Results of these studies are reported below and in Table 4.

**TABLE 4:** Neurotoxicants and Neuropsychological Performance.

**References**	**Group (N)**	**Neuropsychological Tests**	**Strengths**	**Limitations**	**Key Findings and Conclusions**
White et al., 2001	193 GWV, 47 Germany deployed veterans	WAIS-R CPT Trail Making Test PASAT WCST Digit Span CVLT^∗^ WMS-R^∗^ Finger Tapping Purdue Pegboard POMS^∗^ TOMM	– Compared deployed and non-deployed veterans – Detailed account of toxicant exposure – Stratified Random sample	– TOMM scores evidenced possible poor effort in some participants – Multiple comparisons	– Pesticide exposure by self-report was associated with worse mood functioning on all POMS subscales. – Chemical weapons exposure by self-report was associated with worse mood functioning on the POMS subscales of tension and confusion as well as poorer – performance on attention/executive functioning, memory and mood measures.
Sullivan et al., 2003	207 treatment seeking GWV (120 referred for neuropsych evaluation), 53 treatment seeking non-deployed veterans	WAIS-R Information WAIS-R Digit Span Trail Making Test NES CPT Stroop Test Paced Auditory Serial Addition Test Wisconsin Card Sort Test^∗^ CVLT WMS-R Paired Associate Learning WMS-R Visual Reproductions Hooper Visual Organization Test WAIS-R Block Design Finger tapping Purdue Pegboard POMS TOMM	– Investigated deployment, treatment seeking, use of pyridostigmine bromide (PB) and PTSD on cognitive functioning – Matched by control group that was also treatment seeking	– Self-report of exposure – Sample size small for exposure comparisons	– PB use in GWVs showed worse performance on an executive system task. – GWVs with PTSD versus those without PTSD showed no significant differences – There were no significant interaction effects of PB and PTSD on cognitive functioning.
Vasterling et al., 2003	72 GWVs deployed and 33 non-deployed GWVs	WAIS-R Digit Span WCST AVLT CVMT Purdue Pegboard WAIS-R Information	– Selection of a non-treatment seeking group of GWVs – Comparison of deployed GWVs to a group of GWVs mobilized but not deployed – Use of olfactory and neurocognitive measures with demonstrated sensitivity to neurotoxic exposures but not to organophosphates	– Sample was regionally recruited	– No evidence that performance on olfactory or neurocognitive measures were related to self-reported exposure to GW toxicants – GWVs reporting more significant exposures reported greater severity of health symptoms and more severe cognitive symptoms, than those reporting less significant exposures – Symptoms of emotional distress were positively correlated with self-report of health and cognitive complaints
Proctor et al., 2006	140 Army GWV with modeled estimates of nerve agent exposure	CPT Trail Making Test WAIS-R Digit Span WCST Finger Tapping Purdue Pegboard^∗^ WAIS-R Block Design^∗^ CVLT WMS-R verbal paired associate learning WMS visual reproduction	– Stratified random sampling strategy – Examined performance by exposure to sarin and cyclosarin by DOD modeling – Sample was unaware of sarin and cyclosarin components, analyses were conducted prior to exposure knowledge	– Etiology undetermined given the risk of another illness between exposure and measurement (i.e., no baseline health information) – Limited objective information about exposures – Plume estimates only by unit	– Exposure associated with poor fine psychomotor dexterity (*d* = 0.44) and visuospatial abilities (*d* = 0.43) in a dose-response manner – The difference on the motor task was equivalent to the performance effect of being approximately 20 years older and for the block design task, being 15 years older. – Higher exposure was not significantly related to mood state.
Toomey et al., 2009	1061 deployed GWV and 1128 non-deployed GWV	WAIS-III Digit Span Trail Making Test^∗^ PASAT CPT WCST CVLT^∗^ RCFT^∗^ Finger Tapping^∗^ Purdue Pegboard^∗^ TOMM WRAT-III	– Investigated differences in deployment, toxicant exposure, and GWI status – Large sample size, stratified random sampling method – Use of factor analysis – Use of Khamisiyah exposure data	– Low study participation rates from overall larger sample – Cross-sectional design – Neuropsychology raters were not blind to condition – Self-reported PB, pesticide, oil well fire, vaccine exposure	– Those with Khamisiyah exposure modeled sarin exposure showed poor motor speed after controlling for mood – Those reporting proximity to SCUD missiles had lower motor speed – Those reporting CARC paint exposure had worse visual memory – Khamisiyah exposure was associated with poorer verbal memory, beyond emotional distress and demographic variables
Chao et al., 2010	40 GWV with a history of DOD notified sarin cyclosarin exposure risk and 40 non-exposed matched GW veteran controls	CPT Trail Making Test^∗^ WAIS-III Digit Span Short Category Test COWAT^∗^ Grooved Pegboard^∗^ WAIS-III Digit Symbol, matching WAIS-III Block Design^∗^ WAIS-III Verbal Comprehension Index^∗^ CVLT-II WMS-III Logical Memory BVMT-R TOMM	– Use of DOD modeled Khamisiyah data for sarin/cyclosarin exposure – Demographically matched groups	– Lack of information regarding the unit and rank of veterans – Lack of information regarding symptom severity (i.e., CMI, smoking status, head injuries) – Lack of cumulative exposure for all GW veterans – Plume estimates only by unit	– No differences in cognitive measures after controlling for poor effort (i.e., failure of TOMM). – No correlation between unit-level dose-estimates and neuropsychological data in the exposed veterans. – In exposed veterans, hippocampal volume correlate positively with verbal comprehension scores, while total GM volume correlated positively with performance on verbal fluency and visuospatial ability and negatively with time to complete the Trail Making Test and time to place all pegs in the pegboard with the non-dominant hand. – In exposed veterans, total WM volume correlated positively with verbal fluency, and visuospatial function.
Chao et al., 2011	64 sarin and cyclosarin exposed GWVs and 64 “matched” unexposed GWVs	CPT^∗^ WAIS-III Digit Span^∗^ Trail Making Test Short Category Test CVLT-II^∗^ Grooved Pegboard TOMM	– Used matched controls to compare structural and functional differences in veterans with suspected neurotoxicant exposure – Use of more sensitive MRI (4T) – Use of sensitive tests for neuropsychological and mood outcomes	– Lack of information regarding veteran’s unit, severity of GWI symptoms, smoking status, or history of head injury – Neurotoxicant exposure measured at unit over individual level	– Reduced gray matter and white matter in exposed veterans which was linked to sarin/cyclosarin exposure, over and above confounding demographic, clinical, and psychosocial variables. – Exposed veterans made more omission errors and had slower response times on CPT; omission errors was also linked to sarin neurotoxicant exposure – Positive correlation between GM and WM volume, on CVLT performance and digit span backward in the exposed veterans.
Chao et al., 2016	136 GWVs: 106 who reported hearing chemical alarms sound	WAIS III Block Design^∗^ Digit Span^∗^ CVLT		– Had to rely on self-reports of deployment-related exposures – Lack of pre-GW measurements of brain structure and function – Didn’t measure experience and exposure that took place *after* the GW – Small sample size – Lack of a non-deployed GW-era veteran control group	– Self-reported frequency of hearing chemical alarms was inversely associated with and significantly predicted performance on the Block Design visuospatial task. – This effect was partially mediated by the relationship between hearing chemical alarms and lateral occipital cortex volume. – Volumes of the lateral occipital cortex, right inferior frontal cortex, and right supramarginal gyrus were positively correlated with Block design raw scores. – Volumes of lateral occipital cortex, right supramarginal gyrus and right precuneus were negatively correlated with the frequency of hearing chemical alarms. – No dose-effect relationship between Khamisiyah exposures and Block Design raw scores – Frequency of hearing chemical alarms sound was inversely correlated with Backward Digit Span raw scores but not with raw scores on CVLT learning trial 2 or with CVLT short-delay free recall.
Sullivan et al., 2018	159 GW-deployed preventative medicine personnel who had varying levels of pesticide exposure	WAIS-III information subtest Boston Naming Test Trail Making Test^∗^ CPT^∗^ WCST Finger Tapping Grooved Pegboard HVOT RCFT^∗^ Stanford-Binet Copying Test CVLT II POMS^∗^ TOMM	– Grouped veterans by exposure (low/high) to PB and pesticides – Sample had sophisticated knowledge of exposure as they were part of the medical team	– Multiple analyses – Exposures of PB and pesticide may be correlated – Classifications of groups based on self-report	– High pesticide/high PB had worse information processing speed, attention (i.e., errors), visual memory, and increased mood complaints, after controlling for either CMI, PTSD, or depression. – High pesticides/low PB group was the worst performing in terms of visual memory recall while the low pesticides/low PB and low pesticides/high PB group performed significantly better. – Dichlorvos (pest strips) exposure was the best predictor of poorer performance in the attention and psychomotor domains. Methomyl (fly bait) exposure and lindane (delouser) were the best predictors of affective complaints in the mood domain. Bendiocarb and lindane were the best predictors for the visuospatial domain.

*See original journal articles in first column for test references. ^∗^Denotes significance of *p* < 0.05.*

In the Proctor et al. (1998) paper described above, when comparing those exposed or not exposed to self-reported chemical warfare agents, significant differences were found in measures of tension and confusion (POMS), long term visual memory (WMS-R Visual Reproduction), short term verbal memory (CVLT), and attention/working memory (Digit Span). However, those that reported exposure to chemical warfare agents during the war also had lower scores in comparison to the unexposed group. When controlling for mood or malingering, the results did not change, indicating performances were not fully explained by mood disorders and likely represented sequelae from toxicant exposures.

Lindem et al. (2003a) as described above assessed veterans with PTSD and chemical exposure, analyses showed worse performance in sustained attention, motor speed, and motor coordination. Furthermore, researchers concluded that severity of PTSD was a contributing factor to issues with short-term verbal memory (acquisition, retrieval, semantic clustering). Additionally, this pattern suggested difficulties with sustained attention, planning, and executive functioning that may point toward issues with hypervigilance. Finally, self-reported chemical weapons exposure showed specific deficits in sustained attention, perseverative responses, visual memory, and mood measures.

Proctor et al. (2006) examined the relationship between DOD-estimated levels of sarin and cyclosarin exposure and neuropsychological functioning. A stratified random sample of GW veterans completed a medical and history questionnaire, a semi-structured environmental interview, neuropsychological testing, and psychological testing. Veterans were grouped based on exposed vs. non-exposed status as determined by modeled plume exposure estimates obtained from the DoD (from the Khamisiyah weapons depot detonations in March 1991). Results indicated significant differences in groups on psychomotor and visuospatial abilities (e.g., Purdue Pegboard and Block Design) with higher exposure associated with worse outcomes, or a dose response effect with exposure. However, one limitation is the gap between exposure and outcome measurement (4–5 years), thereby making it impossible to determine if it was a delayed or immediate exposure effect. Importantly however, this was the only study conducted before awareness of possible sarin exposure and before DOD notification letters were sent to those exposed at Khamisiyah, Iraq thus reducing bias based on knowledge of exposures.

Toomey et al. (2009) compared 2,000 veterans as described above and additionally performed analyses within the deployed veterans with and without sarin exposure from the Khamisiyah weapons depot detonations as classified by DOD notification (Winkenwerder, 2003). Results showed toxicant exposure was associated with motor speed deficits on CPT over and above mood related effects. In addition, depressive symptoms and exposure to self-reported contaminated food and water were related to worse scores of sustained attention measures. Veterans self-reporting CARC paint exposures had worse visual memory functioning. Veterans reporting being exposed to nerve agents during the war had worse verbal memory functioning and those reporting being near SCUD missiles had lower motor speed.

Chao et al. (2010) investigated neuropsychological performance (White et al., 2001) and MRI results between 40 GW veterans with a history of DOD notified sarin/cyclosarin exposure risk from Khamisiyah and 40 non-exposed matched GW veteran controls. When comparing the controls to the group exposed to sarin/cyclosarin, there were no differences in cognitive measures after controlling for poor effort (i.e., failure on the TOMM). However, the group with sarin exposure had less total gray matter and hippocampal volume on brain imaging. Limitations included lack of information regarding the unit and rank of veterans, lack of information regarding symptom severity (i.e., CMI, smoking status, head injuries), lack of cumulative exposure for all GW veterans, and plume estimates only by unit. Per Janulewicz et al. (2017), there were meaningful effects for Block Design (*d* = −0.32), CPT, reaction time (*d* = 0.42), CVLT long delay (*d* = −0.34), and Pegboard, non-dominant (*d* = 0.27).

Chao et al. (2011) then expanded on these prior findings using a different and larger cohort of veterans (sarin exposed *n* = 65; unexposed controls = 64) and a stronger 4T magnet MRI. Group comparisons on neuropsychological tests showed that sarin exposed veterans had more omission errors and slower reaction time on the CPT. Additionally, there was reduced gray matter and white matter volume in comparison to the control group. Regression analyses also revealed that GWI status was associated with errors of omission, as well as reduced gray matter and white matter volume. Janulewicz et al. (2017) also found meaningful effects in CPT reaction time (*d* = 0.38), Trail Making Test, Part A (*d* = −0.64), Pegboard, dominant (*d* = −0.28), and pegboard non-dominant (*d* = −0.036).

Sullivan et al. (2003) used a sample of 260 veterans including GW deployed and treatment seeking and a control group of GW non-deployed veterans not seeking treatment. Veterans were also compared based on PTSD and the use of pyridostigmine bromide (PB) anti-nerve gas pill usage during the war. All veterans underwent a neuropsychological battery (Table 1) in addition to a structured clinical interview to determine PTSD status. Veterans exposed to PB showed worse performance on a measure of executive system functioning. However, there was no difference between those with and without PTSD on neuropsychological measures and no interaction effect of PB use and PTSD diagnosis. Therefore, the researchers concluded that GW deployment and PB exposure led to the significant neuropsychological decrements.

White et al. (2001) – compared deployed and non-deployed veterans as described above. In this study, pesticide exposure by self-report was associated with worse mood functioning on POMS mood scales. Chemical weapons exposure by self-report was also associated with worse performance on attention/executive functioning, memory and mood measures.

Sullivan et al. (2018) investigated how differing levels of pesticide exposure and PB intake contributed to neuropsychological outcomes in GW veterans. The researchers recruited veterans with functional knowledge of their exposure to neurotoxicants based on their military occupational specialties as military pesticide applicators and/or preventative medical personnel. The four veteran comparison groups were based on pesticide and PB exposures. Participants completed a full neuropsychological test battery (Table 4). Veterans were also assessed for psychological functioning. GWI was determined by CMI criteria (Fukuda et al., 1998). Results showed that high pesticide/high PB exposed group showed significantly slower CPT reaction time and higher POMS symptoms. These neuropsychological decrements remained significant with PTSD as a covariate, demonstrating a main effect on attention reaction time in comparison to the low pesticide/low PB group. Additionally, the high pesticide exposure/low PB group was significantly worse on a measure of visual memory compared to the low pesticide/high PB and low pesticide/low PB groups. Significant differences were found in psychomotor, mood, attention, and memory domains when controlling for covariates (i.e., age, education, gender). Researchers found that a higher rate of CMI was associated with the high pesticide/high PB group which evidenced worse cognitive performance in attention, motor, and memory domains. Overall, results showed that high pesticide/high PB exposure had worse performance on information processing reaction times, attentional errors and visual memory accompanied by increased mood complaints. Limitations of this study include multiple analysis with a smaller sample size, increasing the chance of finding significance. Additionally, it is possible that, although the sample had a sophisticated knowledge of their exposure, their exposures were correlated (i.e., exposure to PB associated with exposure to nerve agents, and pesticides). Additionally, pesticide and PB classifications were reliant on self-report exposure.

Vasterling et al. (2003) compared 72 GW deployed veterans and 33 non-deployed veterans. They compared a full neuropsychological battery and used an olfactory test as a sensitivity measure of toxicant exposure. Results showed no evidence that performance on olfactory or neurocognitive measures were related to war-zone duty or to self-reported exposure to GW toxicants. Symptoms of emotional distress were positively correlated with self-report of health and cognitive complaints. However, the olfactory test has not been shown to be sensitive to organophosphate exposures, the most commonly associated exposure with GWI.

Research on neurotoxicant exposures varied in regard to the toxicant explored (i.e., PB, pesticides or cyclosarin/sarin) and methodology used (i.e., objective or subjective measures). Research would be improved by including more objective biomarkers of past toxicant exposure when comparing deployed and non-deployed troops. Suggested objective biomarkers could be immunological, genetic, or metabolic in nature and would strengthen the link between toxicant exposure and neurological dysfunction given these variables reflect compromised functioning at the time of the study rather than retrospective measures such as self-report or military dose estimate reports. Although markers of past organophosphate exposures have previously been elusive, more recent downstream effects from these exposures have been preliminarily identified and can be utilized (Abou-Donia et al., 2017). Additionally, some studies also addressed investigating illness status or treatment seeking groups (David et al., 2002; Sullivan et al., 2003). Research would improve with more consistent grouping of individuals based on established criteria for GWI (i.e., Steele, 2000). Finally, utilizing recommended CDEs consistently across studies for assessing mood and neuropsychological performance and consistent questions about neurotoxicant exposures would benefit future research as study results would be more comparable.

## Mood and Ptsd Contributions to Neuropsychological Performance

The following five studies that were previously reviewed above concluded that neuropsychological performance was either attributable to mood symptoms or equivalent to controls after accounting for mood-related symptoms (Axelrod and Milner, 1997; Sillanpaa et al., 1997; Storzbach et al., 2000; David et al., 2002; Proctor et al., 2003). Axelrod and Milner (1997) found that GW veterans had elevated scores in a MMPI measure thought to represent body complaints. Sillanpaa et al. (1997), using a model of psychological factors, found that depression (as measured by the MMPI) and anxiety (as measured by the STAI) significantly predicted neuropsychological performance in attention, motor, and executive functioning while an early model of GWI failed to produce significance. Storzbach et al. (2000) found that there was a significant predominately large effect difference in the case group on scores from multiple PTSD and psychological scales. David et al. (2002) found that symptomatic individuals had higher scores on depression, PTSD and anger scales. Finally, Proctor et al. (2003) found that there was significant difference in the POMS mood scales (Fatigue and Confusion) between those reporting GWI symptoms versus controls.

Vasterling et al. (1998) examined GW veterans with (*n* = 19) versus without PTSD (*n* = 24) on measures of attention and memory dysfunction. GW veterans with PTSD performed worse on the WAIS-R Arithmetic test and made more commission errors on the CPT. The GW veterans with PTSD also had worse performance in the Auditory Verbal Learning Test (AVLT) and Continuous Visual Memory Test (CVMT). Vasterling et al. (1998) hypothesized that the presence of intrusions (i.e., inability to inhibit thoughts or experiences related to trauma) could contribute to these patterns of symptoms. Using a principal component analyses, the researchers found that cognitive intrusions symptoms, particularly re-experiencing phenomenon, was related to poorer performance on memory and attention measures (Table 2). Therefore, they hypothesized that PTSD may lead to problems inhibiting inaccurate answers and filtering information unrelated to the task at hand. Of note, the study was limited given that the sample was specifically chosen to have PTSD and they also had other co-morbid diagnoses (i.e., major depression, dysthymia, panic disorder, social phobia, obsessive-compulsive disorder, and somatoform disorder), and included a small sample size making it difficult to control for potential confounds.

In these five studies, there was a lack of consistent use of the same psychological measures making comparing across studies difficult. In addition, some of these psychological tests that measure body complaints and pain, can be interpreted as representing physical or psychological impairments. However, these results showed that depression and PTSD are noteworthy covariates that should be accounted for when investigating neuropsychological performance in GW veterans. Nevertheless, many studies of toxicant exposure and GWI status show neuropsychological deficits even after controlling for mood (Vasterling and Bremner, 2006; Research Advisory Committee on Gulf War Veteran’s Illnesses [RAC-GWVI], 2008). Correspondingly, mood can also be affected by toxicant exposures such as those experienced in the GW indicating another reason why mood should also be assessed in neuropsychological assessments (Sullivan et al., 2018). The recently recommended CDEs for GW research also include measures of PTSD, depression and mood (Table 1).

## Motivation and Malingering Effects on Neuropsychological Functioning

Lindem et al. (2003c) investigated motivation as a contributing factor impacting neuropsychological results in GW veterans. Using a test of malingering and motivation performance validity test (TOMM), the veterans were grouped by those with high scores (> 48) and those with low scores (< 47). Mood related disorders were established using the structured clinical interviews (SCID and CAPS). Results indicated a significant difference on measures of attention, executive functioning, and memory between those with high and low scores on the TOMM. Results also showed some inconsistency across performance given expected patterns with deficits in cognition. Specifically, veterans with lower TOMM scores had lower scores on a verbal memory measure (i.e., Verbal Paired Associates on WMS-R) whilst having higher scores on another test of verbal memory (i.e., CVLT), highlighting the variability that is associated with lower motivation. Additionally, more cognitively challenging items (Trails B, WCST) were more sensitive to low motivation as they required more effort than other tests of simple attention and concentration (Trails A, Digit Span Forward and Backward). Limitations included a small sample of veterans with poor effort (*n* = 18) and a lack of significant clinical measures. The researchers concluded that motivation was an important factor to consider when assessing cognitive performance in GW veterans.

Barrash (2007) proposed that neuropsychological examinations of GW veterans may be unreliable given possible poor effort, invalidating neuropsychological results. A sample of 399 veterans deployed in the GW were divided into three groups: participants without impairments, participants exhibiting impairment with credible results or participants with impaired and non-credible results. Participants underwent a full neuropsychological battery with results adjusted based on age, gender, and estimated premorbid intellect. In addition, veterans completed measures assessing psychological functioning and subjective cognitive complaints. Malingering was measured using the Exaggeration Index of the AVLT (Rey, 1964), Recognition Memory Test (RMT), performance across cognitive domains, error types, and MMPI-2 validity indices. Researchers found lower levels of non-credible performance among GW veterans, and those with non-credible validity results had worse impairment on nearly all neuropsychological tests. In addition, those in the non-credible group were more likely to endorse worse subjective cognitive symptoms and emotional and social impairment. Therefore, Barrash (2007) concluded that non-credible results are relatively rare in GW populations (<1%). In addition, researchers found consistently worse performance patterns in non-credible profiles indicating that a malingering measure should be used in neuropsychological assessments. Barrash (2007) noted some limitations including a small sample of the non-credible group, decreased statistical power, and a lack of measures indicating the reason for poor effort.

Both of these studies show a lower rate than expected in GW veterans for malingering or lowered motivation performances. However, it was demonstrated that low effort can lead to worse outcomes on neuropsychological testing as well as variable test performances that makes it difficult to interpret the true cognitive profile of GW veterans with low effort. Therefore, it is recommended that all neuropsychological batteries include measures of motivation and malingering that can be used as covariates in analyses or where those performing sub-optimally on these measures can be removed from data analyses. An element of the recent CDEs for neuropsychological assessment includes a motivational measure (CVLT Forced Choice, Table 1).

## Discussion

Gulf War illness is a CMI, impacting the health of a significant amount of GW veterans; however, the etiology and treatment of GWI remains somewhat elusive, prompting the demand for more research. Research investigating the neuropsychological underpinnings of GWI is especially needed given the prevalence of cognitive symptoms in GW veterans, possibly the second most reported symptom in GWI (Smith et al., 2012).

Early studies of neuropsychological functioning and GW veterans focused more on the etiology of these symptoms with conflicting results pointing either toward a mood related or neurological cause. These studies did not use an established criterion and compared groups based on their deployment status (deployed, non-deployed) and/or symptom presence (reporting symptoms, not reporting symptoms). Therefore, early review papers such as Axelrod and Milner (2000) recommended that further research should use more testable operational definitions of GWI (see Table 2).

Despite the efforts to establish criteria for GWI, researchers continued to find mixed results on the etiology of GWI centering on the debate of a mood related or neurotoxicant underpinning or both. Vasterling and Bremner (2006) highlighted that the impact of mood and the discrepancy between subjective reports and objective measurements made it more difficult to determine the etiology of any deficits observed. Further studies controlled for mood effects in analyses (i.e., PTSD or depression), however, different outcomes and case criteria used continued to make clear comparisons across studies difficult to interpret. For example, David et al. (2002) found substantial evidence of mood related nature of GWI using the Fukuda et al. (1998) CDC criteria in United Kingdom veterans,. Wallin et al. (2009) expanded on these findings using the CDC criteria in United States veterans and only found differences in GWI on depression, somatic complaints, and anxiety and were underpowered to detect neuropsychological impairments. Further research using the Haley GW syndromes eluded to more physiological causes (Hom et al., 1997). Nevertheless, focusing on these criteria or the presence of GW-related symptoms did not necessarily clarify the etiology of GWI. However, no study to date has compared the IOM and Kansas case criteria when comparing neuropsychological outcomes. Because the Kansas criteria has been shown to be a more specific case criteria than other measures used in prior studies (CDC, ME/CFS, ‘slow’ cases), this may provide more clarity with regard to neuropsychological impairment profiles in veterans with GWI.

In addition, there is now consistent evidence across nine papers comparing neurotoxicant exposures and neuropsychological outcomes (see Table 4). Seven out of the nine studies found significant neuropsychological differences when comparing exposures through either sarin/cyclosarin, organophosphate pesticides or PB anti-nerve gas pills. These studies point toward a pattern of neurotoxicant exposure and neurocognitive decline given their relative similarity to other occupationally exposed groups of agricultural workers or pesticide applicators (Ismail et al., 2012; Mackenzie Ross et al., 2013). Therefore, neurotoxicant exposures may have an impact on particular neuropsychological domains including attention, executive system, memory and motor functioning as a result of chemicals that impact acetylcholine inhibition and induce neuroinflammation (Sullivan et al., 2003, 2018; Proctor et al., 2006; Toomey et al., 2009).

Additional research focused on other pertinent topics in GWI including subjective memory and effort. Briefly, one of the three studies found a correlation between subjective memory and objective memory functioning. Future research would benefit from consistency of subjective and objective cognitive queries.

As PTSD can be a relevant comorbidity in GW veterans, this review also included research investigating PTSD and GW veterans in relation to neuropsychological performance. In two out of three smaller studies, veterans with PTSD who had served in the GW showed worse performance in verbal memory (i.e., working memory, intrusions, recognition, and interference), visual memory, general intellectual ability, sustained attention, motor speed, and visuospatial skills (Vasterling et al., 1998, Lindem et al., 2003a). However, these findings were not replicated in a larger study of treatment seeking veterans (Sullivan et al., 2003).

The GW was a short combat mission of just 4 days of actual ground war, resulting in a PTSD prevalence rate of less than 10% of GW veterans in population-based studies (Research Advisory Committee on Gulf War Veteran’s Illnesses [RAC-GWVI], 2008). Although studies of convenience samples with self-referred participants generally have higher PTSD rates. Prevalence rates of comorbidity between those with GWI and PTSD need to be fully investigated. Given the significant overlap of comorbidity in convenience sample studies, treatment studies should consider comorbid therapies where both disorders can be treated at the same time.

Correspondingly, GWI and stress or PTSD symptoms as a comorbid condition is supported by neuroinflammation and HPA axis research. O’Callaghan et al. (2015) and Koo et al. (2018) found that stress, as induced via corticosterone exposure, extended the impact of neuroinflammation from diisopropyl fluorophosphate (i.e., DFP sarin surrogate), in a rat model of GWI. This suggests that the effect of the neurotoxicant is worsened by the stressor rather than the cause. Furthermore, O’Callaghan et al. (2015) identified cytokine biomarkers (i.e., TNF-alpha, interleukin 6) which were more highly elevated after corticosterone exposure. Additionally, they found that treatment with anti-inflammatory antibiotic minocycline reduced the inflammatory response. Koo et al. (2018) found that DFP exposure was associated with wide spread microstructural integrity changes on diffusion imaging in the thalamus, amygdala, piriform cortex, and ventral tegmentum area whereas the rats also treated with corticosterone had more restricted patterns within the hypothalamus and hippocampus. Ashbrook et al. (2018) supported this finding identifying epigenetic biomarkers or transcriptional histone modification and DNA methylation in genes possibility linked to neuroinflammation and cognition in a rat model of GWI with DFP and corticosterone.

In research with human participants, Golier et al. (2012) found that GW veterans with PTSD were more likely to have higher plasma adrenocorticotropic hormone after exposure to corticotropin-releasing factor. Additionally, this change was associated with higher exposure to PB. Golier et al. (2012) concluded that this reflects HPA dysfunction in GW veterans. Furthermore, Golier et al. (2016) found that treatment via mifepristone was associated with improvements in verbal learning in GW veterans with CMI which was mediated by cortisol change levels suggesting some overlap with cognitive functioning and HPA axis health.

Suggestions for future research include using measures consistent across studies. Additionally, future research should continue to utilize more objective measures of neurological dysfunction (i.e., imaging, genetic studies, immunological factors) in conjunction with recommended neuropsychological and psychological test measures. These studies show the importance of controlling for PTSD and other mood effects when comparing neuropsychological outcomes in veterans with GWI. Although research is varied on GWI and its sub components (i.e., PTSD, effort, and subjective reporting), there remains strong evidence of neuropsychological decrements. The etiology of these results remains unclear but has been further linked to neurological dysfunction.

Mood factors remain relevant given their potential to exacerbate neurological dysfunction by possibly proliferating neuroinflammation (O’Callaghan et al., 2015; Koo et al., 2018). Despite these efforts, the heterogenous nature of methodologies investigating neuropsychological deficits limits the ability to truly identify the etiology of the neuropsychological decline without instituting common data elements of core tests used in all future neuropsychological studies. However, research has been improved to support evidence of GWI leading to deficits measurable through neuropsychological batteries, particularly in areas including attention, memory, motor functioning, and executive functioning. Notable improvements include the use of established criteria and measuring toxicant exposure especially through objective biomarkers (Abou-Donia et al., 2017). However, future research would benefit from continuing to use established criteria when investigating neuropsychological performance in GWI. Linking objective biomarkers with neuropsychological outcomes could provide potential markers for treatment development. For instance, research on cytokine profiles of GWI have shown immunological homeostatic shifts, which lends credence to a neurological etiology in GWI and treatment avenues to pursue (Golier et al., 2012; Craddock et al., 2015; Abou-Donia et al., 2017). Finally, there has been a lack of research investigating GWI and PTSD comorbidity.

## Conclusion

In conclusion, neuropsychological research in GWI has improved in methodology but continues to leave questions regarding the etiology and cognitive difficulties in veterans. Future research should utilize improved methods with the use of standardized methodology and assessment batteries, increase measurement sensitivity and increase consistency while expanding into additional realms of study (i.e., immunological and neuroimaging biomarkers) that could further explain the underlying pathobiology of GWI. Through this research, clinicians can utilize sensitive neuropsychological instruments which in turn will more effectively inform treatment efficacy for the multitude of veterans impacted by GWI and its co-morbidities.

Therefore, the neuropsychological CDEs (Gulf War Illness Research Program [GWIRP], 2019) is an excellent resource for identifying highly recommended measures for future research to allow direct comparison of study results. It is also imperative that these measures are adapted for use in imaging studies to understand the functional and structural underpinnings of cognitive impairments and changes over time in this aging group of veterans who are at higher risk for chronic medical conditions (Zundel et al., 2019).

Our recent meta-analysis of neuropsychological studies found impairments in visuospatial, attention, executive function, and learning and memory domains which were found in three or more prior studies (Janulewicz et al., 2017). This literature review supports these findings while considering the impact of neurotoxicant and mood factors. Future research assessing treatments or investigating biomarkers of GWI should include neuropsychological outcomes in the domains of visuospatial, attention and executive function, and learning and memory. Specifically, tests to include are Block Design, Trail Making Test, Digit Span, and CVLT, as these are known sensitive measures in GW veterans. For the clinician, GW veterans are an aging population at higher risk for chronic medical conditions and therefore their subjective cognitive complaints should be documented and evaluation with the sensitive neuropsychological measures recommended in this review.

## Author Contributions

MJ, JK, NK, MK, KS, and TC contributed to the conception and design of the review. MJ, CZ, and TC compiled review materials. MJ wrote the first draft of the manuscript. JK, NK, MK, CZ, KS, and TC wrote sections of the manuscript. All authors contributed to manuscript revision, read and approved the submitted version.

## Conflict of Interest

The authors declare that the research was conducted in the absence of any commercial or financial relationships that could be construed as a potential conflict of interest.

## Footnotes

1 https://cdmrp.army.mil/gwirp/research_highlights/19gwi_cde_initiative_highlight.aspx
